# Microbial Diversity Analysis of Soil in the Rhizosphere of *Securidaca longipedunculata* (African Violet Tree)

**DOI:** 10.3390/microorganisms13112636

**Published:** 2025-11-20

**Authors:** Sphelele Zondi, Mahloro Hope Serepa-Dlamini, Pfariso Maumela

**Affiliations:** Department of Biotechnology and Food Technology, Faculty of Science, University of Johannesburg, Doornfontein Campus, P.O. Box 17011, Doornfontein, Johannesburg 2028, South Africa; sphelelerajesh@gmail.com (S.Z.); hopes@uj.ac.za (M.H.S.-D.)

**Keywords:** biocontrol, biochemical, biofertilizer, fungicidal, polymerase chain reaction

## Abstract

This study explored the microbial diversity within the rhizosphere of *Securidaca longipedunculata* (African violet tree), a medicinal plant recognized for its ethnobotanical importance. Six rhizospheric bacterial isolates were identified and characterized for their plant growth-promoting abilities and environmental resilience. Growth-promoting assays demonstrated that the isolates could grow in a nitrogen free environment, solubilize phosphate, produce ammonia, and synthesize indole acetic acid (IAA). Morphological and biochemical characterizations differentiated four Gram-positive from two Gram-negative strains. The bacterial isolates demonstrated plant-growth promoting potential, showing an enhanced ability (*p*-value < 0.05) to promote root elongation and biomass accumulation compared to the control treatments. The strains showed antifungal properties with some isolates recording 100% fungal mycelial growth and spore germination inhibition. Phylogenetic studies linked these isolates to the genera *Pseudomonas* and *Bacillus*. These findings highlight the diversity of rhizospheric bacteria associated with *S. longipedunculata* and emphasize their role in enhancing soil fertility and plant resilience to pathogens.

## 1. Introduction

*Securidaca longipedunculata* (African violet tree) is a medicinal plant species from the family *Polygalaceae*, under the genus *Securidaca* [1]. *S. longipedunculata* is a multipurpose medicinal plant due to its extensive use in African traditional medicine [2]. The roots, stem, and leaves of the African violet tree are used for treating a wide range of ailments [3]. The plant is used to treat ailments such as fever, pain, epilepsy, pneumonia, tuberculosis, venereal diseases, syphilis, and coughs, and as an aphrodisiac [4].

The medicinal value of *S. longipedunculata*, is attributed to its diverse phytochemical composition of bioactive compounds [5,6]. Phytochemical studies have revealed that different parts of the plant including the leaves, fruits, stem bark, seeds, and roots contain a variety of bioactive compounds. These compounds include xanthones, benzyl benzoates, triterpenes, alkaloids, phenols, flavonoids, terpenoids, anthraquinones, and saponins, that possess different pharmacological properties [5,7]. The bioactive compounds have been shown to possess antioxidants, antitumor, anti-inflammatory, analgesic, antimicrobial, and hepatoprotective activities [5,7]. *S. longipedunculata* currently faces endangerment and extinction due to its heightened demand and exploitation [8] for medicinal applications [9]. The unsustainable harvesting practices, without adequate regeneration of the species, have consequently precipitated a marked decline in the wild population of *S. longipedunculata* [10]. Considering these challenges, it is imperative to promote sustainable harvesting techniques and enforce conservation measures to mitigate the impact of over exploitation [11,12].

Moreover, medicinal plants have been reported to have symbiotic relationships with soil microorganisms that are beneficial to the host plant [13]. The plant thus relies on these beneficial bacteria, known as rhizobacteria, for nutrient availability and protection against pathogens and pests. Rhizobacteria can rapidly colonize the rhizosphere, producing biologically active compounds, such as antibiotics, antifungal agents, and siderophores, that suppress soil-borne pathogens [14]. The microbes also induce systemic resistance in plants, priming the host defense against a broad range of potential infections [15]. Moreover, plants have been reported to potentially transfer specific genes to rhizosphere bacteria, enhancing the bacteria’s ability to produce compounds that are vital for plant defense [16]. Horizontal gene transfer (HGT) between plants and bacterial endophytes or rhizospheric bacteria could allow plants to co-opt bacterial metabolic pathways, and rely on bacterial efficiency in synthesizing bioactive compounds [17]. This genetic exchange gives plants an evolutionary advantage by harnessing the rapid growth and high metabolic output of bacteria [18]. This potential gene transfer mechanism could explain the heightened efficacy with which some rhizobacteria secrete secondary metabolites that deter pathogens or promote plant growth [17].

Therefore, understanding the microbial diversity of rhizobacteria and their interactions with host plants will enable researchers to harness bacterial biosynthetic capabilities to address challenges in medicine and agriculture. Rhizobacteria are a sustainable alternative for the large-scale production of biologically active products. Furthermore, bacteria, with their fast replication cycles and efficient metabolic processes, can be genetically engineered to synthesize biologically active compounds with biotechnological potential. Thus, the aim of this study was to characterize the microbial diversity in the rhizosphere of *S. longipedunculata* using biochemical tests and genomic tools. Moreover, their ability to support host plant growth and confer protection against microbial pathogens was evaluated through in vivo and in vitro experiments.

## 2. Materials and Methods

### 2.1. Sample Collection and Preparation

Soil samples, along with associated plant materials, were collected in Musina (−22° 24′ 59.99″ S, 29° 44′ 59.99″ E), Limpopo Province, South Africa. The soil sample was obtained from a depth of 15–20 cm within the rhizosphere of *S. longepedunculata* (African violet tree). The sample was immediately wrapped in plastic, to preserve its integrity and stored in a sealed container. It was then transported under controlled conditions to the University of Johannesburg, where it was stored at 4 °C, until further processing, to prevent microbial activity and preserve the native microbial communities present in the sample.

### 2.2. Isolation of Rhizospheric Microorganisms

A total of 1 g of the soil sample was transferred into a sterile Falcon tube containing 9 mL of sterile saline solution (0.9% *w*/*v*). The mixture was agitated in a shaking incubator (Labcon Model 5082U, Labcon, Krugersdorp, South Africa) at 300 rpm for 15 min to dislodge microbial cells from the soil particles. The sample was subsequently allowed to settle briefly. A serial dilution was performed to reduce the microbial load for isolation purposes. Dilutions ranging from 10^−1^ to 10^−5^ were prepared using sterile distilled water. A 0.5 mL aliquot was aseptically transferred from the 10^−3^ to 10^−5^ dilutions and plated on nutrient agar. The plating was carried out in duplicates to enhance reproducibility. The plates were incubated at 28 °C for 24–48 h, allowing for the growth of microbial colonies and the emergence of distinct colonies.

### 2.3. Colony Selection and Biochemical Characterization

#### 2.3.1. Colony Selection

A total of 15 colonies were selected based on their distinct morphological characteristics, including shape, size, color, and texture, from the microbial colonies that emerged on nutrient agar plates. The colonies were then subcultured onto fresh nutrient agar plates to obtain pure cultures. During the subculturing process, some colonies were identified as fungal contaminants, reducing the initial number of colonies from 15 to 6. Bacterial colonies that exhibited uniform growth were selected for further analysis.

#### 2.3.2. Catalase Activity

Biochemical profiles were used to confirm the physiological diversity and enzymatic capabilities of the isolates. The catalase activity was determined by adding 100 µL of 30% hydrogen peroxide (H_2_O_2_) to 100 µL fresh overnight bacterial culture. The catalase activity was qualitatively monitored by observing a sudden effervescent reaction, which indicated the enzymatic decomposition of H_2_O_2_ into water and oxygen [19].

#### 2.3.3. Urease Test

The urease test was performed in Christensen’s urea agar. The basal medium components (1 g of peptone, 5 g of NaCl, 1 g of dextrose, 2 g KH_2_PO_4_, 0.012 g of phenol red and 15 g of agar in 900 mL of water) were suspended in water and sterilized in an autoclave. The urea concentrate (20 g of urea in 100 mL) was prepared by dissolving urea in water and filter sterilizing. Then, 100 mL of the urea concentrate was aseptically added to 900 mL of the cooled basal medium. Christensen’s urea agar was added to sterile tubes that were subsequently slanted at 2 cm. A fresh overnight bacterial culture was streaked on the slanted surfaces and tubes incubated at 30 ± 2 °C overnight. A color change from yellow to pink confirmed the hydrolysis of urea into ammonia and carbon dioxide [20].

#### 2.3.4. Methyl Red Test

The methyl red test assessed the production of stable acids from glucose fermentation. The methyl red–Voges Proskauer (MR–VP) broth components (7 g of buffered peptone, 5 g of glucose and 5 g of K_2_HPO_4_ in 1 L of water) were suspended in water and sterilized by autoclaving. Then, 5 mL of the cooled broth was aseptically aliquoted into sterile tubes. Methyl red solution (0.02%) was prepared by dissolving 0.1 g of methyl red in 300 mL of 95% ethyl alcohol. Distilled water was added to make up a final volume of 500 mL. A loop full of a fresh overnight culture was added to the MR–VP broth and incubated at 28 ± 2 °C for 48 h. Five drops of methyl red indicator were then added after incubation. A persistent red coloration signified a positive result [21].

#### 2.3.5. Oxidase Activity

The oxidase activity was evaluated by adding a drop of the oxidase reagent (1% tetramethyl-p-phenylenediamine dihydrochloride) onto an overnight fresh culture on a nutrient agar plate. The formation of a dark purple coloration within 10 s indicates a positive reaction [22].

### 2.4. Determination of Plant Growth-Promoting Properties: In Vitro

#### 2.4.1. Growth in Nitrogen Free Environment

The ability of the bacterial isolates to grow in a nitrogen free environment was performed according to the method of Jensen (1954) [23]. Jensen’s medium components (20 g of sucrose, 1 g of K_2_HPO_4_, 0.5 g of MgSO_4_, 0.5 g of NaCl, 0.1 g of FeSO_4_, 0.005 g of Na_2_MoO_4_, 2 g of CaCO_3_, and 15 g of agar in 1 L of distilled water) were suspended in distilled water, autoclaved, and agar poured into plates after cooling. The plates were subsequently streaked with the bacterial isolates and incubated at 28 ± 2 °C for 2–3 days. Bacterial growth on the nitrogen-free Jensen’s medium indicated the successful ability of the isolates to grow in a nitrogen free environment.

#### 2.4.2. Phosphate Solubilization

The ability of the isolates to solubilize inorganic phosphate was assessed using the method of Pikovskaya (1948) [24]. Pikovskaya’s (PVK) medium components (5 g of yeast extract, 10 g of dextrose, 5 g of Ca_3_(PO_4_)_2_, 0.5 g of (NH_4_)_2_SO_4_, 0.2 g of KCl, 0.1 g of MgSO_4_, 0.0001 g of MnSO_4_, 0.0001 g of FeSO_4_ and 15 g of agar in 1 L of distilled water) were suspended in water and autoclaved to sterilize the medium. The agar was cooled and poured into plates, under sterile conditions. The bacterial isolates were streaked onto the prepared PVK plates and incubated at 28 ± 2 °C for 5 days. The formation of a clear halo region around the bacterial colonies indicated phosphate solubilization activity.

#### 2.4.3. Indole Acetic Acid (IAA) Production

The ability of the isolates to produce indole acetic acid was determined using the method of Sarwar and Kremer (1995) [25]. The tryptone broth components (10 g of tryptone, 3 g of beef extract, 5 g of NaCl, and 0.204 g of L-tryptophan in 1 L of distilled water, pH 7) were suspended in water, pH adjusted to 7 and sterilized by autoclaving. The cooled medium was aseptically aliquoted into 5 mL volumes in glass test tubes. Then, 50 µL of each bacterial isolate was inoculated into the 5 mL medium and incubated in a shaking incubator (Labcon Model 5082U, Labcon, Krugersdorp, South Africa) at 180 rpm and 28 ± 2 °C for 3 days. After incubation, 1.5 mL of the culture was centrifuged at 12,000 rpm for 10 min. Then, 1 mL aliquot of the supernatant was mixed with 1 mL of sterile Salkowski reagent (50 mL of 35% perchloric acid and 1 mL of a 0.5 M FeCl_3_ solution). The mixture was incubated in the dark for 1 h. The development of a red or faint pink coloration indicated the presence of IAA.

#### 2.4.4. Ammonia Production

Ammonia production was assessed using the qualitative method of Ahmad et al. (2008) [26]. Peptone water components (10 g of peptone, 5 g of NaCl, 7 g of Na_2_HPO_4_, and 3 g of KH_2_PO_4_ in 1 L of distilled water) were suspended in water and sterilized by autoclaving. Then, 20 µL of an overnight bacterial culture of the test strain was inoculated into 10 mL peptone water and incubated for three days in a shaking incubator (Labcon Model 5082U, Labcon, Krugersdorp, South Africa) at 180 rpm and 28 ± 2 °C. After incubation, 0.5 mL of sterile Nessler’s reagent (3 g of KI and 3 g of HgI_2_ dissolved in 20 mL of distilled water) was added to each culture. A color change from brown to yellow showed ammonia production.

### 2.5. DNA Extraction and Polymerase Chain Reaction (PCR) Amplification

The Zymo DNA Extraction Kit (Zymo Research) was used to extract genomic DNA from the bacterial isolates. The universal bacterial primers 27F (forward) and 1492R (reverse) were utilized for the 16S rRNA gene sequencing for bacterial identification and phylogenetic analysis. Then, 2 µL of the isolated DNA was combined with 23 µL of the PCR reaction mixture. The PCR mixture consisted of 12.5 µL of Taq Master Mix (New England Biolabs, Ipswich, MA, USA), 6.5 µL of nuclease-free water, and 2 µL each of the forward and reverse primers (Inqaba Biotech, Pretoria, South Africa). The Taq Master Mix provided the necessary components for DNA amplification, including DNA polymerase, dNTPs, and reaction buffers. Each DNA template underwent single-pass sequencing using the 16S rRNA universal primers (27F and 1492R). The PCR reaction was performed in a thermocycler (Biorad Model T100 Biorad, Sandton, South Africa). The reaction conditions were denaturing, annealing, and extension at 95, 48, and 72 °C, respectively, with 35 cycles. The resulting sequences were used for the identification of the bacterial isolates and phylogenetic analysis using MEGA 11.

### 2.6. Antifungal Assays

#### 2.6.1. Mycelial Growth Inhibition Assay

The six bacterial isolates were tested for their potential fungicidal activity against the agronomically significant fungal phytopathogen *Botrytis cinerea*. A *B. cinerea* strain was cultured on potato dextrose agar (200 g/L potato infusion, 20 g/L glucose and 20 g/L agar) plates for 3 days at 25 °C. Fresh mycelial plugs were subsequently collected from the colony edge and placed on a new potato dextrose agar (PDA) plate that had been plated with 150 µL of an overnight culture (~1 × 10^6^ CFU per mL) of the test bacterial strain. Negative control PDA plates were plated with 150 µL of sterile nutrient broth. The experiments were conducted in triplicate. The cultures were incubated at 25 °C and mycelial growth inhibition determined after 3 days. The relative inhibition of mycelium growth was quantified as [(C − T)/C] × 100, where T is the diameter of the treated sample and C is the diameter of the negative control.

#### 2.6.2. Spore Germination Inhibition Assay

A 10-day old culture of *B. cinerea* on PDA was used for the harvesting of spores using a saline solution. Then, 10 µL of spores with a concentration of 10^5^ spores/mL were spot inoculated to a fresh PDA plate that had been plated with 150 µL of an overnight culture (~1 × 10^6^ CFU per mL) of the test bacterial strain. The control treatment was plated with 150 µL of sterile nutrient broth. The experiments were conducted in triplicate. The plates were incubated at 25 °C and examined after 3 days for spore germination/growth inhibition. The relative germination inhibition (RGI, %) of spores was quantified as [(C − T)/C] × 100, where T is the spore germination rate of the treated sample and C is the spore germination rate of the control sample.

### 2.7. In Vivo Plant Growth Promotion Assays

Tomato seeds were germinated in a seedling tray. The seedlings were individually transferred and planted in plant pots with potting soil. An overnight bacterial culture of the test strain was centrifuged at 8000 rpm for 10 min. The pellet was resuspended in water to a concentration of 1 × 10^6^ CFU per mL. Then, 1 mL of the test bacterial strain, suspended in water, was then added to each pot (seedling); water was used as the control. The seedlings were allowed to grow for 10 days after which the root length, fresh root, and shoot weights were determined. Eight replicates were performed for each test strain.

### 2.8. Statistical Analysis

Antifungal and plant growth assays were performed in replicate. The analysis of variance was performed in IBM SPSS Statistics Version 25.0 at a *p*-value of 0.05 with Tukey’s post hoc tests. The graphs were drawn in Microsoft Excel 2025 version.

## 3. Results

### 3.1. Isolation, Morphological and Biochemical Characterization of Bacterial Endophytes

The serial dilution plating technique, for the isolation of rhizospheric bacteria, resulted in the identification of 15 colonies based on their distinct morphology. However, the number was subsequently reduced to six bacterial strains through the exclusion of fungal contaminants. The six selected bacterial strains were further subcultured to obtain uniform colonies. From the six bacterial strains, it was determined that four strains were Gram-positive, while two were Gram-negative (Table 1). The bacterial strains were further characterized using biochemical tests based on their ability to synthesize and secrete certain enzymes [27]. The isolated bacterial strains all tested positive for the methyl red, catalase, and oxidase test. However, only two of the six strains showed a positive outcome for the urea test.

### 3.2. Plant Growth Promotion: In Vitro

**Figure 1:**
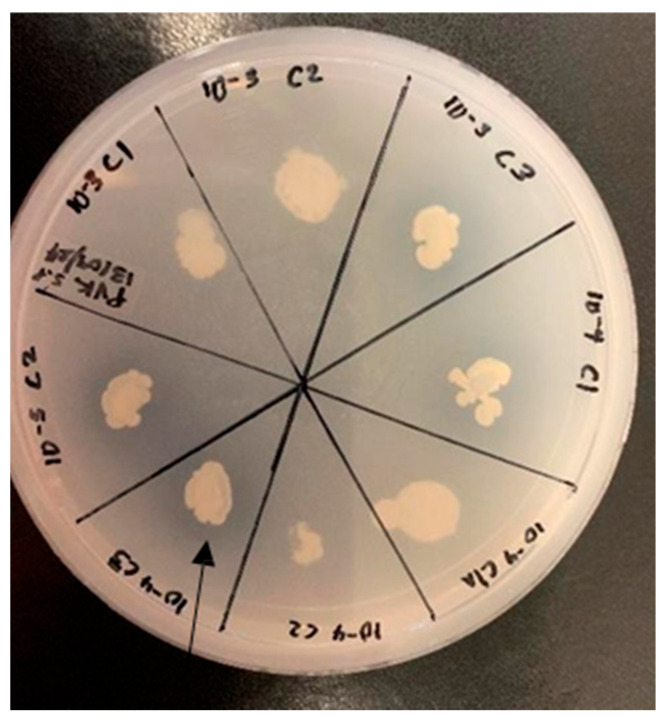
Phosphate solubilization of the selected bacterial strain on PVK agar.

The six bacterial isolates were evaluated, in vitro, for their plant growth-promoting traits, including phosphate solubilization, growth in nitrogen free environment, ammonia production, and indole acetic acid (IAA) production. Using Pikovskaya’s (PVK) agar method, isolates 10^−3^ C3, 10^−4^ C1, and 10^−4^ C3 exhibited significantly larger zones of phosphate solubilization compared to other test strains (Figure 1). Isolates 10^−3^ C1, 10^−3^ C3, 10^−4^ C1, and 10^−4^ C3 demonstrated an ability to grow in a nitrogen free environment. Ammonia production was assessed qualitatively using Nessler’s reagent, with all bacterial isolates showing positive results for ammonia solubility (Table 2). In addition, isolates 10^−4^ C1 and 10^−4^ C3 exhibited the ability to produce indole acetic acid.

### 3.3. The Role of Rhizospheric Bacteria in Antifungal Activity and In Vivo Plant Growth Promotion

**Figure 2:**
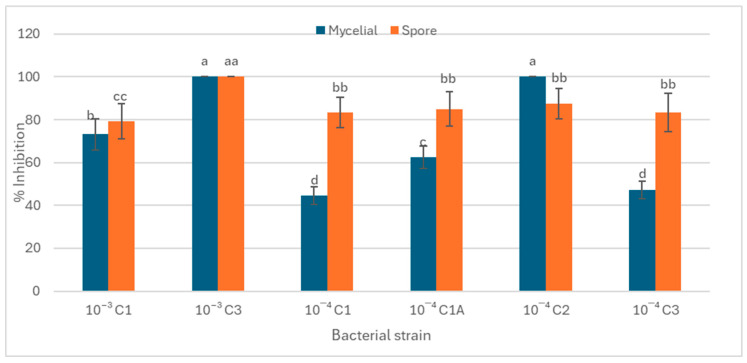
Inhibition of mycelial growth and spore germination by rhizospheric bacterial isolates from *S. longipedunculata*. The antifungal activity was assessed on PDA plates at 25 °C. Error bars represent the standard error. Distinct letters/symbols for the same treatment indicate statistically significant differences.

**Figure 3:**
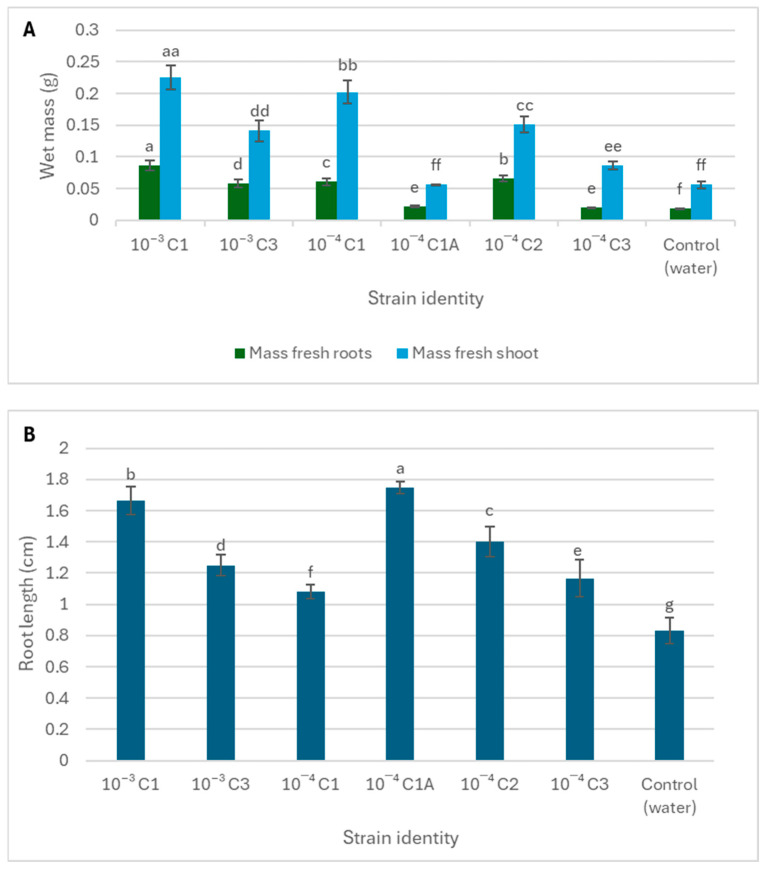
In vivo PGP assay treating tomato seedlings with rhizospheric bacterial isolates from *S. longipedunculata* to determine the impact on the fresh root and stem mass (**A**) and root length (**B**). The seeds were allowed to germinate and grow for 10 days. The error bar represents the standard error. Different letters/symbols for the same treatment indicate statistically significant differences.

Figure 2 and Figure 3 demonstrate a dual functional role of the rhizospheric bacterial isolates from *S. longipedunculata* in both pathogen suppression and plant growth enhancement. Strains such as 10^−3^ C3, and 10^−4^ C2 displayed significantly higher (>80%) fungicidal activity against both active (mycelial) and dormant (spore) forms of *B. cinerea* (Figure 2). Furthermore, the rhizospheric bacteria displayed traits associated with plant growth promotion, however, the correlation was not always direct between fungicidal activity and plant growth promotion. For example, strain 10^−3^ C1 demonstrated moderate fungal growth inhibition of 73.22% but significantly enhanced seedling biomass, particularly the fresh shoot mass (Figure 3). The lowest mycelial growth inhibition was recorded in isolate 10^−4^ C1 and 10^−4^ C3. On the other hand, strain 10^−4^ C1A, which had strong antifungal effects, primarily promoted root elongation with a minimal effect on shoot biomass. Isolate 10^−4^ C1A only demonstrated the potential to promote root elongation.

### 3.4. Phylogenetic Analysis

**Figure 4:**
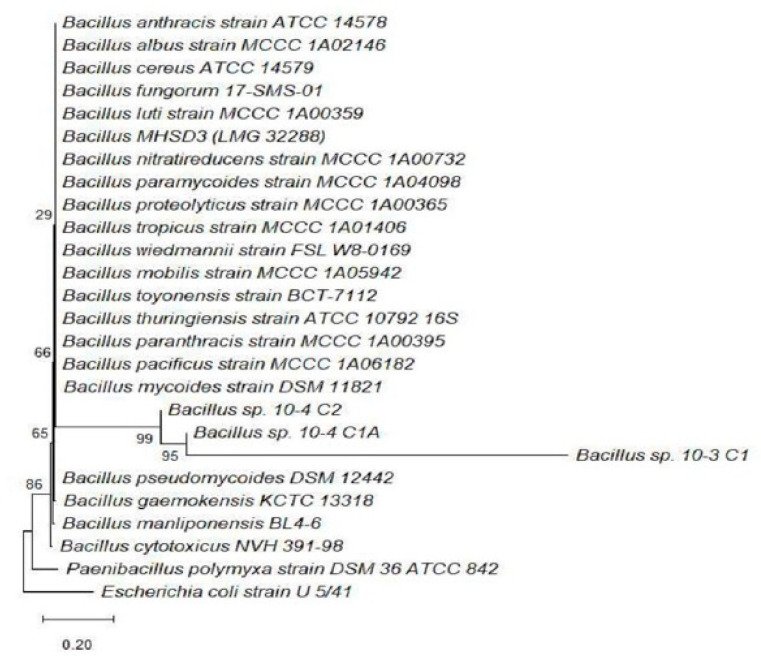
Composite phylogenetic tree of bacterial isolates (10^−3^ C1, 10^−4^ C1A, and 10^−4^ C2) constructed using the Neighbor-Joining method in MEGA 11. The tree is rooted and includes bootstrap values at key nodes.

**Figure 5:**
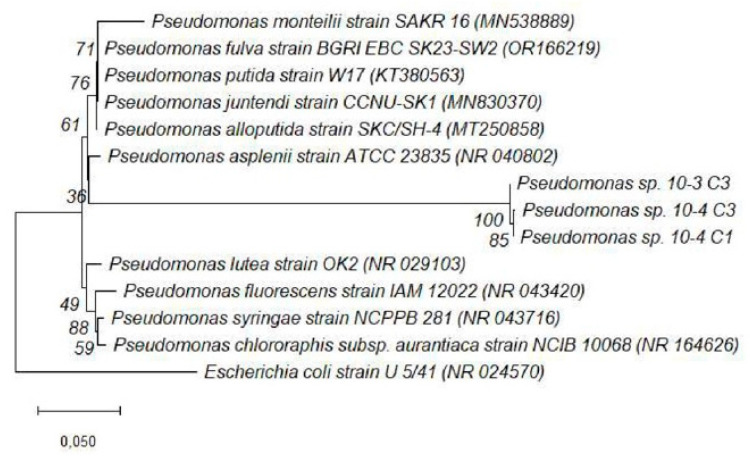
Composite phylogenetic tree of bacterial isolates (10^−3^ C3, 10^−4^ C1, and 10^−4^ C3) constructed using the Neighbor-Joining method in MEGA 11. The tree is rooted and includes bootstrap values at key nodes.

Phylogenetic analysis based on 16S rRNA gene sequences was conducted to determine the evolutionary relationships of the bacterial isolates (10^−3^ C1, 10^−3^ C3, 10^−4^ C1, 10^−4^ C1A, 10^−4^ C2, and 10^−4^ C3), with known reference strains. The Neighbor-Joining method implemented in MEGA 11 was used to construct rooted trees, allowing for the clear resolution of clades and lineage divergence (Figure 4 and Figure 5). The use of bootstrapping (1000 replicates) provided confidence in the branching topology.

## 4. Discussion

The morphological and biochemical properties of the rhizospheric bacterial, isolated from the African violet tree, are consistent with rhizobacteria isolated from the rhizosphere of other medicinal plants [28,29]. Singh et al. [29] reported that bacterial endophytes from the medicinal plant *Azadirachta indica* were both Gram-positive and -negative rod-shaped isolates. Jabborova et al. [24], also reported the dominance of Gram-positive and -negative rod-shaped rhizospheric bacteria associated with medicinal plants. The mutual relationship between rhizospheric bacteria and their host plant is sustained by the ability of the former to promote plant growth and offer plant protection to their host [14]. The production of catalase by rhizospheric bacteria has been reported to regulate plant growth and antioxidative defense systems in cotton plants [30]. Rhizospheric bacteria enhances nutrient availability for host plants through the hydrolysis of urea into ammonia, which can be used as a nitrogen source by the plants [31]. The experimental data consequently demonstrated the presence of rhizospheric bacteria from *Securidaca longipedunculata*, which are capable of indole acetic acid and ammonia production (Table 1). Furthermore, rhizospheric bacteria such as strain 10^−3^ C3, 10^−4^ C1, and 10^−4^ C3 showed the ability of phosphate solubilization (Figure 1).

Strains such as 10^−3^ C3, and 10^−4^ C2 displayed consistent and complete inhibition of *B. cinerea* conidial mycelial growth. Thus, this strong fungicidal activity makes the strains have a potential biotechnological application in biocontrol. The findings suggest the presence of potent bioactive secondary metabolites capable of interfering with fungal spore germination or mycelial growth pathways through mechanisms such as cell wall degradation or the inhibition of energy metabolism [32]. Some of these anti-fungal strains also exhibited traits associated with plant growth promotion [33]. For example, strain 10^−3^ C1 demonstrated moderate antifungal activity (73.22% inhibition) but significantly enhanced seedling biomass, particularly the fresh shoot mass. This may be attributed to a strong ability of strain 10^−3^ C1 to produce metabolites such as IAA or ammonia that preferentially stimulate vegetative growth [34]. The lowest inhibition was recorded in isolates 10^−4^ C1 and 10^−4^ C3, indicating limited antagonistic potential against *B. cinerea* [35]. The variability observed among isolates may be attributed to differences in their ability to synthesize antifungal metabolites, produce lytic enzymes, or compete effectively for nutrients and space [36,37].

The phylogenetic tree (Figure 4) of isolates 10^−3^ C1, 10^−4^ C1A, and 10^−4^ C2, shows that the strains formed a well-supported clade within the genus *Bacillus*. These isolates clustered closely with *Bacillus pseudomycoides*, *Bacillus gaemokensis*, and other related species, supported by bootstrap values exceeding 80% [38]. The position of isolate 10^−4^ C1A within the *Bacillus cereus* group is of particular interest, as this clade includes species known for their robust stress resistance and the production of antimicrobial compounds [39]. These *Bacillus*-affiliated isolates are spore-forming, a trait that enhances their survival in diverse soil conditions, thus making them promising candidates for biocontrol applications [40].

In contrast, Figure 5 depicts the placement of isolates 10^−3^ C3, 10^−4^ C1, and 10^−4^ C3 within the *Pseudomonas* genus. These isolates formed a distinct clade with *Pseudomonas fluorescens*, *Pseudomonas alloputida*, and related strains. The positioning of isolate 10^−4^ C3 revealed a slightly divergent lineage within the cluster, hinting at the possibility of a novel environmental strain. The strong bootstrap values supporting these branches underscore the reliability of the taxonomic assignments [38]. *P. fluorescens* and *P. alloputida* are commonly associated with rhizospheric environments, where they contribute to plant health through multiple mechanisms such as siderophore production, phosphate solubilization, and antifungal activity [41]. Their placement in the tree correlates with the functional traits observed in this study, such as high IAA production and consistent antifungal activity [42]. The rooted nature of both trees was essential in confirming the evolutionary divergence between *Bacillus* and *Pseudomonas* lineages, further validating the dual-genus distribution of the isolates [38]. This phylogenetic separation not only reinforces the distinct functional niches these bacteria occupy in the rhizosphere but also highlights their complementary roles in promoting plant health [42]. While *Bacillus* species are known for their spore-forming ability and enzyme production, *Pseudomonas* spp. possess rapid colonization and secondary metabolite synthesis traits [40]. The observed data in this study illustrate the diversity of rhizospheric bacteria associated with *S. longipedunculata* and their potential biotechnological application.

The phylogenetic findings aligned well with the BLAST version 2.17.0 results, where most isolates shared ≥98% identity with their closest type strains [38]. This taxonomic clarity provides a foundation for further characterization and the potential commercial application of the bacterial isolates [43]. Furthermore, the congruence between phylogenetic groupings and phenotypic traits such as IAA production, phosphate solubilization, and antifungal activity indicates that the observed evolutionary relationships have potential functional consequences [44]. The phylogenetic data confirm that isolates from the rhizosphere of *S. longipedunculata* belong predominantly to the *Bacillus* and *Pseudomonas* genera, both of which are recognized for their ecological versatility and plant-beneficial roles [45]. These findings support the broader aim of identifying rhizospheric bacteria with potential application in sustainable agriculture and the conservation of medicinal plant ecosystems

## 5. Conclusions

This study characterized rhizospheric bacteria associated with *S. longipedunculata*, highlighting their roles in plant growth promotion and pathogen suppression. Isolates from *Bacillus* and *Pseudomonas* showed traits entailing phosphate solubilization, potential nitrogen fixation, and antifungal activity against *B. cinerea*. Their dual function as fungicidal agents and plant growth promoters illustrates their potential biotechnological applications in agriculture. Findings also support conservation efforts for *S. longipedunculata* through microbial symbioses, proposing future research on metabolite profiling and genome mining for enhanced formulations. The variability among strains emphasizes the need for targeted selection and necessitates additional research applying genomic and metabolomic approaches for further characterization. This will result in the identification and characterization of specific metabolites of biotechnological significance. This work enhances our understanding of plant–microbe relationships and native rhizobacteria’s role in ecological restoration and potential applications in sustainable crop production.

## Figures and Tables

**Table 1:** Morphological and biochemical characterization of bacterial isolates from the rhizosphere of *Securidaca longipedunculata*.

Selective bacterial strains						
Characteristics						
	10^−3^ C1	10^−3^ C3	10^−4^ C1	10^−4^ C1A	10^−4^ C2	10^−4^ C3
Morphological characterization						
Gram Staining	Positive	Negative	Negative	Positive	Positive	Positive
Shape	Rod	Rod	Rod	Rod	Rod	Rod
Form	Round	Round	Round	Round	Round	Round
Surface	Smooth	Shiny	Smooth	Smooth	Smooth	Smooth
Color	Creamy white	Creamy white	Creamy white	Creamy white	Creamy white	Creamy white
Size	Medium	Small	Small	Medium	Medium	Small
Elevation	Entire	Entire	Entire	Entire	Entire	Entire
Opacity	Opaque	Opaque	Opaque	Opaque	Opaque	Opaque
Biochemical characterization						
Catalase	+	+	+	+	+	+
Urea	−	+	−	−	−	+
Methyl Red	+	+	+	+	+	+
Oxidase	+	+	+	+	+	+

“−“ indicates absence, “+” presence positive, “C” indicates the colony number, and “A” indicates the new distinct colony that resulted from the already selected colony.

**Table 2:** In vitro plant growth promoting assays of selected bacterial isolates from the rhizosphere of *Securidaca longipedunculata*.

Strain	Phosphate Solubilization	Growth in Nitrogen Free Environment	Ammonia Solubilization	IAA Production
**10** **^−3^ C1**	−	+	+	−
**10** **^−3^ C3**	+	+	+	−
**10** **^−4^ C1**	+	+	+	+
**10** **^−4^ C1A**	−	−	+	−
**10** **^−4^ C2**	−	−	+	−
**10** **^−4^ C3**	+	+	+	+

“+” Indicates presence, “−” absence.

## Data Availability

The original data (the 16S rRNA sequences) presented in the study are openly available in the NCBI GenBank database at SUB1576539.

## Abbreviations

The following abbreviations are used in this manuscript:

IAA	Indole acetic acid
HGT	Horizontal gene transfer
PDA	Potato dextrose agar
PDB	Potato dextrose broth
DNA	Deoxyribonucleic acid
RNA	Ribonucleic acid
PVK	Pikovskaya
PGP	Plant growth promoting
PCR	Polymerase chain reaction
dNTPs	Deoxynucleotide triphosphates
RGI	Relative germination inhibition

## References

[1] Lijalem T., Feyissa T. (2020). In vitro propagation of *Securidaca longipedunculata* (Fresen) from shoot tip: An endangered medicinal plant. J. Genet. Eng. Biotechnol..

[2] Shai K., Lebelo S.L., Manyeula F., Mabelebele M., Sebola A.N. (2025). Assessment of *Securidaca longipedunculata* (violet tree) effect on Semen Quality and Blood Sex Hormone Levels in Indigenous Goats. Vet. Anim. Sci..

[3] Fomnya H.J., Ngulde S.I., Saka S., Gana S.M., Umaru B., Auwal M.S. (2023). Anticonvulsant and anxiolytic activities of methanol extract of *Securidaca longipedunculata* Fres. root bark in mice. J. Appl. Pharm. Sci..

[4] Daboué E.M., Béné A., Dimobe K., Dayamba D.S., Zouré A.B., Ouattara B., Sabo P., Tuina S., Neya O., Vinceti B. (2024). Variability in fruit morphology and germination capacity of the tropical medicinal species *Securidaca longipedunculata* Fres. Seeds.

[5] Mongalo N.I., McGaw L.J., Finnie J.F., Van Staden J. (2015). *Securidaca longipedunculata* Fresen (Polygalaceae): A review of its ethnomedicinal uses, phytochemistry, pharmacological properties and toxicology. J. Ethnopharmacol..

[6] Shah M., Mubin S., Hassan S.S.U., Tagde P., Ullah O., Rahman M.H., Al-Harrasi A., Rehman N.U., Murad W. (2022). Phytochemical profiling and bio-potentiality of genus scutellaria: Biomedical approach. Biomolecules.

[7] Neelu Joshi N.J., Alok Shukla A.S., Nailwal T.K. (2014). Taxonomic and phytomedicinal properties of *Oroxylum indicum* (L.) Vent: A wonderful gift of nature. J. Med. Plants Res..

[8] Abubakar U.S., Khalifa B.I., Abdu F., Sanusi M., Gawuna T.A., Adamu J.G., Rogo S.S. (2018). Threatened medicinal plants of Kano flora and the need for urgent conservation. Int. J. Conserv. Sci..

[9] Obode O.C., Adebayo A.H., Omonhinmin C.A., Yakubu O.F. (2020). A systematic review of medicinal plants used in Nigeria for hypertension management. Int. J. Pharm. Res..

[10] Saranraj P., Sivasakthi S., Deepa M.S. (2016). Phytochemistry of pharmacologically important medicinal plants—A review. Int. J. Curr. Res. Chem. Pharm. Sci..

[11] Chen S.L., Yu H., Luo H.M., Wu Q., Li C.F., Steinmetz A. (2016). Conservation and sustainable use of medicinal plants: Problems, progress, and prospects. Chin. Med..

[12] Chen G., Sun W. (2018). The role of botanical gardens in scientific research, conservation, and citizen science. Plant Divers..

[13] Wang G., Ren Y., Bai X., Su Y., Han J. (2022). Contributions of beneficial microorganisms in soil remediation and quality improvement of medicinal plants. Plants.

[14] Tzipilevich E., Russ D., Dangl J.L., Benfey P.N. (2021). Plant immune system activation is necessary for efficient root colonization by auxin-secreting beneficial bacteria. Cell Host Microbe.

[15] Meena M., Swapnil P., Divyanshu K., Kumar S., Harish, Tripathi Y.N., Zehra A., Marwal A., Upadhyay R.S. (2020). PGPR-mediated induction of systemic resistance and physiochemical alterations in plants against the pathogens: Current perspectives. J. Basic Microbiol..

[16] Berendsen R.L., Vismans G., Yu K., Song Y., de Jonge R., Burgman W.P., Burmølle M., Herschend J., Bakker P.A., Pieterse C.M. (2018). Disease-induced assemblage of a plant-beneficial bacterial consortium. ISME J..

[17] Giron D., Dedeine F., Dubreuil G., Huguet E., Mouton L., Outreman Y., Vavre F., Simon J.C. (2017). Influence of microbial symbionts on plant–insect interactions. Advances in Botanical Research.

[18] Lyu D., Msimbira L.A., Nazari M., Antar M., Pagé A., Shah A., Monjezi N., Zajonc J., Tanney C.A., Backer R. (2021). The coevolution of plants and microbes underpins sustainable agriculture. Microorganisms.

[19] Boufares K., Kouadria M., Karima M., Merdjet Y.N. (2023). Investigating the effects of plant growth-promoting rhizobacteria isolates on germination and physiology status of durum wheat under salt stress. Acta Agric. Slov..

[20] Lehman D.C. (2014). Gram-Negative Bacteria. Textbook of Diagnostic Microbiology-E-Book: Textbook of Diagnostic Microbiology-E-Book.

[21] Bangun A. (1981). Taxonomic Study of Pasteurella Anatipestifer.

[22] Oakey H.J., Ellis J.T., Gibson L.F. (1996). A biochemical protocol for the differentiation of current genomospecies of Aeromonas. Zentralblatt Bakteriol..

[23] Jensen H. (1954). The azotobacteriaceae. Bacteriol. Rev..

[24] Pikovskaya R.I. (1948). Mobilization of Phosphorus in Soil in Connection with Vital Activity of Some Microbial Species. Microbiology.

[25] Sarwar M., Kremer R.J. (1995). Determination of bacterially derived auxins using a microplate method. Lett. Appl. Microbiol..

[26] Ahmad F., Ahmad I., Khan M. (2008). Screening of free-living rhizospheric bacteria for their multiple plant growth promoting activities. Microbiol. Res..

[27] Lanyi B. (1988). 1 Classical and rapid identification methods for medically important bacteria. Methods in Microbiology.

[28] Jabborova D., Mamarasulov B., Davranov K., Enakiev Y., Bisht N., Singh S., Stoyanov S., Garg A.P. (2024). Diversity and plant growth properties of rhizospheric bacteria associated with medicinal plants. Indian J. Microbiol..

[29] Singh A.K., Sharma R.K., Sharma V., Singh T., Kumar R., Kumari D. (2017). Isolation, morphological identification and in vitro antibacterial activity of endophytic bacteria isolated from *Azadirachta indica* (neem) leaves. Vet. World.

[30] Verma P., Hiremani N.S., Gawande S.P., Sain S.K., Nagrale D.T., Narkhedkar N.G., Prasad Y.G. (2022). Modulation of plant growth and antioxidative defense system through endophyte biopriming in cotton (*Gossypium* spp.) and non-host crops. Heliyon.

[31] Rana K.L., Kour D., Kaur T., Sheikh I., Yadav A.N., Kumar V., Suman A., Dhaliwal H.S. (2020). Endophytic microbes from diverse wheat genotypes and their potential biotechnological applications in plant growth promotion and nutrient uptake. Proc. Natl. Acad. Sci. India Sect. B Biol. Sci..

[32] Feofilova E.P., Ivashechkin A.A., Alekhin A.I., Sergeeva Y.E. (2012). Fungal spores: Dormancy, germination, chemical composition, and role in biotechnology. Appl. Biochem. Microbiol..

[33] Poorter H., Niklas K.J., Reich P.B., Oleksyn J., Poot P., Mommer L. (2012). Biomass allocation to leaves, stems and roots: Meta-analyses of interspecific variation and environmental control. New Phytol..

[34] Jan M., Muhammad S., Jin W., Zhong W., Zhang S., Lin Y., Zhou Y., Liu J., Liu H., Munir R. (2024). Modulating root system architecture: Cross-talk between auxin and phytohormones. Front. Plant Sci..

[35] Podgórska-Kryszczuk I., Solarska E., Kordowska-Wiater M. (2022). Biological control of *Fusarium culmorum*, *Fusarium graminearum* and *Fusarium poae* by antagonistic yeasts. Pathogens.

[36] Duncan N., Horta A., Conallin J., Marsden T., Lynch A.J., Stuart I. (2025). Reframing Fish Passage Prioritization for Human Nutrition Outcomes. Environ. Manag..

[37] Wang H., Liu R., You M.P., Barbetti M.J., Chen Y. (2021). Pathogen biocontrol using plant growth-promoting bacteria (PGPR): Role of bacterial diversity. Microorganisms.

[38] Christensen H., Olsen J.E.D. (2023). Introduction to Phylogenetic Analysis of Molecular Sequence Data. Introduction to Bioinformatics in Microbiology.

[39] Farina D., Bianco A., Manzulli V., Castellana S., Parisi A., Caruso M., Fraccalvieri R., Serrecchia L., Rondinone V., Pace L. (2024). Antimicrobial and Phylogenomic Characterization of *Bacillus cereus* Group Strains Isolated from Different Food Sources in Italy. Antibiotics.

[40] Goraj W., Szafranek-Nakonieczna A., Grządziel J., Polakowski C., Słowakiewicz M., Zheng Y., Gałązka A., Stępniewska Z., Pytlak A. (2021). Microbial involvement in carbon transformation via CH_4_ and CO_2_ in saline sedimentary pool. Biology.

[41] Rajkumar M., Bruno L.B., Banu J.R. (2017). Alleviation of environmental stress in plants: The role of beneficial *Pseudomonas* spp. Crit. Rev. Environ. Sci. Technol..

[42] Leitão F., Alves M., Henriques I., Pinto G. (2025). Endophytic Bacterial Consortia Isolated from Disease-Resistant *Pinus pinea* L. Increase Germination and Plant Quality in Susceptible Pine Species (*Pinus radiata* D. Don). Forests.

[43] Suman A., Govindasamy V., Ramakrishnan B., Aswini K., SaiPrasad J., Sharma P., Pathak D., Annapurna K. (2022). Microbial community and function-based synthetic bioinoculants: A perspective for sustainable agriculture. Front. Microbiol..

[44] Cherif-Silini H., Silini A., Yahiaoui B., Ouzari I., Boudabous A. (2016). Phylogenetic and plant-growth-promoting characteristics of Bacillus isolated from the wheat rhizosphere. Ann. Microbiol..

[45] Kour D., Kour H., Khan S.S., Khan R.T., Bhardwaj M., Kailoo S., Kumari C., Rasool S., Yadav A.N., Sharma Y.P. (2023). Biodiversity and functional attributes of rhizospheric microbiomes: Potential tools for sustainable agriculture. Curr. Microbiol..

